# ZnO Thin Films as Promising Corrosion Protection on Mg-Based Alloys

**DOI:** 10.3390/ma18245568

**Published:** 2025-12-11

**Authors:** Aneta Kania, Magdalena M. Szindler, Marek Szindler, Zbigniew Brytan, Monika Kciuk, Wojciech Pakieła, Łukasz Reimann, Paweł M. Nuckowski

**Affiliations:** 1 Department of Engineering Materials and Biomaterials, Faculty of Mechanical Engineering, Silesian University of Technology, Konarskiego 18a, 44-100 Gliwice, Poland; magdalena.szindler@polsl.pl (M.M.S.); zbigniew.brytan@polsl.pl (Z.B.); monika.kciuk@polsl.pl (M.K.); wojciech.pakiela@polsl.pl (W.P.); 2 Scientific and Didactic Laboratory of Nanotechnology and Material Technologies, Faculty of Mechanical Engineering, Silesian University of Technology, Towarowa 7, 44-100 Gliwice, Poland; marek.szindler@polsl.pl; 3 Materials Research Laboratory, Faculty of Mechanical Engineering, Silesian University of Technology, Konarskiego 18a, 44-100 Gliwice, Poland; lukasz.reimann@polsl.pl (Ł.R.); pawel.nuckowski@polsl.pl (P.M.N.)

**Keywords:** ZnO thin films, ALD method, structure analysis, topography analysis, corrosion studies

## Abstract

The present study examined the microstructure and corrosion characteristics of MgCa4Zn1Gd1 and MgCa2Zn1Gd3 alloys that were coated with ZnO thin films, which were deposited by atomic layer deposition (ALD). Coatings of different thicknesses (42.5, 95.4 and 133.7 nm for 500, 1000, and 1500 cycles, respectively) were characterized using X-ray diffraction (XRD), Raman spectroscopy, SEM/EDS, AFM (atomic force microscope), and FTIR (Fourier transform infrared spectroscopy). XRD and Raman analyses were conducted to verify the formation of crystalline zinc oxide (ZnO) with a homogeneous granular morphology. Surface roughness decreased with increasing coating thickness, reaching the lowest values for the 1500-cycle ZnO layer on MgCa2Zn1Gd3 (R_a_ = 7.65 nm, R_s_ = 9.8 nm). Potentiodynamic and immersion tests in Ringer solution at 37 °C revealed improved corrosion resistance for thicker coatings, with the lowest hydrogen evolution (20.89 mL·cm^−2^) observed for MgCa2Zn1Gd3 coated after 1500 cycles. Analysis of corrosion products by FTIR identified Mg(OH)_2_ and MgCO_3_ as dominant and then MgO and ZnO. Phase analysis also indicated the presence of ZnO coating after 100 h of immersion. The ZnO film deposited after 1500 ALD cycles on MgCa2Zn1Gd3 provides the most effective corrosion protection and is a promising solution for biodegradable magnesium implants.

## 1. Introduction

The utilization of orthopedic biomaterials serves to stabilize, protect, replace, and regenerate damaged musculoskeletal tissues. In recent times, biodegradable materials have gained considerable popularity due to their capacity to decompose. The utilization of these biomaterials eliminates the need for additional surgical intervention to remove implant materials, as they undergo degradation within the human body [1]. The use of magnesium (Mg) and Mg alloys as temporary implants has the potential to significantly reduce healthcare costs and the risk of infections acquired during subsequent hospitalizations. Mg-based alloys possess numerous favorable properties for orthopedic applications, including low density, high specific strength, and biocompatibility. It is unfortunate that these materials also exhibit low corrosion resistance and a high degradation rate in solutions containing chlorine, such as those found in human body fluids. As a result, excess hydrogen is released in the vicinity of the wound after the implantation procedure [2]. The corrosion of magnesium may result in local alkalinization at the surface of magnesium alloy implants and a subsequent decrease in mechanical strength in implants with a prolonged degradation period [1,2,3,4,5,6]. It is therefore evident that the application of suitable coatings or protective layers, obtained through surface treatment, is essential for magnesium alloys when they are used in environments where they may come into contact with the human body. The surface properties of the alloy or coated alloy should facilitate osseointegration and degradation, while ensuring that the rate at which this occurs is consistent with the rate of tissue growth. Furthermore, these materials are suitable for a diverse range of applications, including those within the field of orthopedics.

The use of zinc oxide (ZnO) as a coating or layer applied to biodegradable alloys is a topic of significant interest. However, it should be noted that ZnO used internally is only absorbed by the body to a limited extent. Zinc is an essential element for human health [7]. It plays a role in enzyme synthesis, hormone production, and protein and nucleic acid metabolism. Additionally, Zn is a potent antioxidant that delays the aging process, improves collagen metabolism, and regenerates the skin. Zinc is a vital nutrient that is essential for optimal growth and reproduction. Zinc oxide has been demonstrated to possess astringent, drying, and antibacterial properties. In a study by Sun et al. [8], a ZnO coating was synthesized on an AZ31 magnesium alloy by microwave aqueous synthesis and subsequent heat treatment. Subsequently, the coating was irradiated with ultraviolet light and immersed in a simulated body fluid (SBF) solution, with the objective of enhancing its biological and antibacterial activity. The presence of carbonate-containing hydroxyapatite was detected on the surface of the magnesium alloy that had been coated. The results of the in vitro antibacterial experiment demonstrated that the ZnO sample that had been exposed to UV rays and immersed in SBF for two weeks exhibited excellent inhibition of bacterial cell growth. This was evidenced by an antibacterial index of 94.50 ± 1.25% against S. aureus and 98.95 ± 0.71% against E. coli, which was significantly higher than that observed for the uncoated Mg alloy. In the work [6], the addition of the ZnO film effectively reduced the corrosion activity of the AZ31B alloys when exposed to body fluids. The open circuit potential (E_OCP_) for the ZnO-coated alloy measured in SBF was more stable and was located higher than the curve for uncoated AZ31B. In addition, the corrosion potential (E_corr_) values for the uncoated AZ31B and the AZ31B with a ZnO film were −0.575 and −0.45 V, respectively. The corrosion current density (j_corr_) values for AZ31B and AZ31 with a ZnO layer were 1.365 × 10^−4^ A·cm^−2^ and 1.297 × 10^−4^ A·cm^−2^, respectively. In the article [9], a nanostructured coating of silver-doped zinc oxide (Ag-ZnO) was deposited on the Mg-2Ca-0.5Mn-6Zn alloy using the PVD (physical vapor deposition) method. Researchers determined that the applied coating exhibits a reduced corrosion rate (as evidenced by a lower corrosion current density) in comparison to the uncoated alloy. Additionally, antimicrobial testing demonstrated a markedly enhanced antimicrobial capacity against Escherichia coli and Staphylococcus aureus for the coated alloy compared to the Mg alloy substrate. In other research, Bakhsheshi-Rad et al. [10] deposited ZnO and ZnO/Ca_3_ZrSi_2_O_9_ coatings on the surface of a MgCaZn alloy using PVD and EPD (electrophoretic deposition) techniques. The results of the corrosion tests demonstrated that the samples coated with zinc oxide and ZnO/Ca_3_ZrSi_2_O_9_ exhibited enhanced corrosion resistance and superior stability in a simulated body fluid when compared to the uncoated samples. Furthermore, the antibacterial activity of the samples was evaluated using the disk diffusion method. The results demonstrated that the deposited ZnO and ZnO/Ca_3_ZrSi_2_O_9_ coatings exhibited significant inhibitory effects against *E. coli*, *Klebsiella*, and *Shigella*. Furthermore, the potential of nano-sized zinc oxide in medical applications is significant due to its antibacterial and anticancer properties. The material has been shown to have wound-healing properties (that is, it can be used as an astringent) and has been found to be an effective treatment for eczema and hemorrhoids [11].

The objective of this study is to examine the surface morphology and topography of thin ZnO layers deposited by the ALD (atomic layer deposition) method on Mg-based alloys (MgCa4Zn1Gd1 and MgCa2Zn1Gd3) with different concentrations of calcium and gadolinium. A significant element of the research involves the assessment of the corrosion resistance of the layers during potentiodynamic and immersion testing, along with the measurement of H_2_ release. The authors will examine the surface morphology and topography before corrosion testing in a chlorine-rich Ringer’s environment and the surface morphology and corrosion products after corrosion tests.

## 2. Materials and Methods

The MgCa4Zn1Gd1 and MgCa2Zn1Gd3 alloys were selected for the deposition of thin ZnO films due to their properties as substrate materials. The preparation of the Mg-based alloys was carried out using high-purity metals: magnesium (Mg, 99.99%, Stanchem, Niemce, Poland), calcium (Ca, 99.5%, Pol-Aura, Zabrze, Poland), zinc (Zn, 99.99%, Gliwenturex, Gliwice, Poland), and gadolinium (Gd, 99.9%, Thermo Fisher Scientific, Waltham, MA, USA). The alloys were cast in an induction furnace at a temperature of 750 °C with argon serving as protective gas. For the ALD process, samples were prepared in the form of cylinders with diameters of 13 mm and heights of 7 mm. All samples were subjected to mechanical polishing with SiC paper (Struers, Ballerup, Denmark), grade 500–4000, and subsequently with a diamond suspension. Ultrasonic degreasing was performed for a duration of 10 min, followed by cleaning with alcohol and washing with distilled water.

Zinc oxide (ZnO) thin films were synthesized on magnesium-based alloys utilizing an ALD Picosun R-200 reactor (Picosun, Espoo, Finland). Diethylzinc served as the metal precursor, while deionized water was employed as the oxidizing agent. The thermal parameters of the atomic layer deposition were applied for selected materials, and the deposition process was conducted at a temperature of 300 °C. The pulse durations for the precursor and water were set at 0.1 s and 4 s, respectively. Between each precursor and reagent pulse, a 4 s nitrogen (N_2_) purge step was implemented to eliminate residual precursors and reaction by-products. The deposition cycles for zinc oxide (ZnO) thin films ranged from 500 to 1500 cycles.

The thickness of the resulting thin films was evaluated using an FR-pRo-UV/VIS optical reflectometer (ThetaMetrisis SA, Peristeri, Greece), which operates based on the principle of light reflection. This method relies on the phenomenon of total reflection to determine the thickness by analyzing the ratio of reflected to incident light. When the light beam strikes the film surface, partial reflections occur at both the upper and lower interfaces of the film, generating interference. This interfered signal is transmitted via optical fiber to a CCD array, where it is recorded and processed. The resulting output is a spectrogram exhibiting interference fringes, which are directly related to the thickness of the deposited film and are displayed on the monitor. The film thickness was measured at five locations for each sample, and the standard deviation (SD) reflects the statistical variation in these measurements. The resulting values were: 500 cycles: 42.5 nm ± 2.1 nm, 1000 cycles: 95.4 nm ± 4.3 nm, 1500 cycles: 133.7 nm ± 6.2 nm. The inclusion of these uncertainties reflects the surface roughness of the ZnO layers and provides a more accurate representation of the film thickness variation.

### 2.1. Structure and Phase Analysis

Morphological observations of the samples were characterized using a scanning electron microscope (SEM, SUPRA 35 model, Zeiss, Thornwood, New York, NY, USA; EHT = 5.0 kV, 15 kV, in-lens detector, BSD detector), which was equipped with an energy-dispersive X-ray spectroscopy detector (EDS). EDS analysis was used to identify the surface and the resulting corrosion products after corrosion tests.

Surface topography observations and roughness measurements were carried out using a Park Systems (Park Systems, Suwon, Republic of Korea) AFM XE-100 atomic force microscope. The experiment was conducted in non-contact mode. The observation area was 25 µm^2^. The XEI software version 1.8 was used to calculate the roughness parameters (e.g., roughness average (R_a_) and root mean square (R_s_)).

Phase analysis of the ZnO thin films was performed using a PANalytical X’Pert PRO X-ray diffractometer (Malvern Panalytical, Almelo, The Netherlands) with Co Kα radiation. Analysis was performed using the step registration method over a 2θ angular range of 30–90°. Qualitative X-ray analysis was performed using HighScore Plus 3.0e version software and a dedicated PAN-ICSD (Inorganic Crystal Structure Database) phase identification card database.

The identification of the ZnO layer structure was also confirmed using a Renishaw Raman spectrometer (Renishaw, model inVia Reflex, New Mills, UK) equipped with an Ar ion laser operating at 514.5 nm. The spectra were recorded in the wide spectral range of 150–3200 cm^−1^ with a spectral resolution of approximately 1 cm^−1^. The measurements were carried out in backscattering geometry under ambient conditions.

### 2.2. Corrosion Study—Immersion Tests

The corrosion resistance studies encompassed electrochemical and immersion tests. Electrochemical studies were conducted using an Atlas 0531EU and IA potentiostat (Atlas-Sollich, Gdansk, Poland). The measurements were conducted in Ringer solution, which consists of 8.6 g·dm^−3^ NaCl, 0.3 g·dm^−3^ KCl, and 0.48 g·dm^−3^ CaCl_2_·6H_2_O at 37 °C. The corrosion potential scan rate was 1 mV·s^−1^. The polarization curves with Tafel extrapolation were determined. The stabilization time was 5 min. After that, the corrosion parameters, e.g., the corrosion potential, E_corr_; the corrosion current density, j_corr_; and the corrosion polarization resistance, R_p_, were determined. Potentiodynamic studies were performed on three samples in each case. The results of the Tafel analysis were presented for a representative sample.

The immersion tests of the samples were performed in Ringer solution at a temperature of 37 °C for a duration of 48 and 100 h. The measurements obtained provided an estimate of the gas corrosion product (H_2_ evolution volume). For the measurements, cylindrical samples with a testing area of 1.3 cm^2^ were prepared. The volume of evolved H_2_ was then measured in relation to the frontal area of the samples.

Potentiodynamic and immersion studies were performed on three samples in each case. The results of the Tafel analysis and the volume of H_2_ released were presented for a representative sample.

### 2.3. Analysis of Corrosion Products

Following immersion testing, the corroded surfaces of the MgCa4Zn1Gd1 and MgCa2Zn1Gd3 alloys with ZnO layers were observed by SEM, with the EDS analysis of the corrosion products providing valuable additional insights. Furthermore, the corrosion products were analyzed using an X-ray diffractometer (Malvern Panalytical, Almelo, The Netherlands) with Co Kα radiation. The analysis was performed using the step registration method. The angular range was 10–110°. Qualitative XRD analysis was performed using HighScore Plus 3.0e version software and a dedicated PAN-ICSD phase identification card database.

The composition of the corrosion products was also confirmed using Fourier transform infrared (FTIR) spectroscopy. The FTIR spectra of the MgCa4Zn1Gd1 and MgCa2Zn1Gd3 alloys with the ZnO thin films were recorded at room temperature using a Nicolet 6700/8700 FTIR spectrometer (Thermo Fisher Scientific, Waltham, MA, USA). For the purpose of this study, corrosion products were collected from the surface of immersed samples and then amalgamated with dry KBr. The samples were conducted in transmission mode within a mid-infrared range of 4000 to 400 cm^−1^.

## 3. Results and Discussion

**Figure 1:**
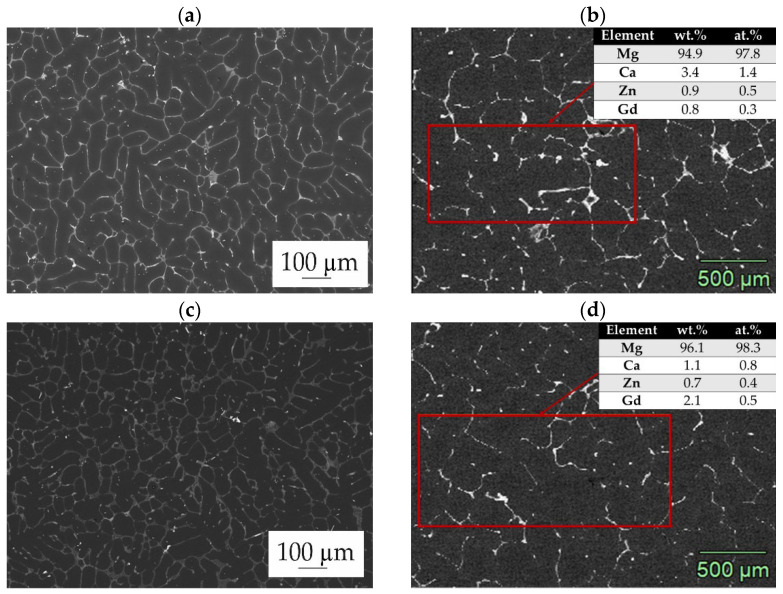
Microstructure of (**a**,**b**) MgCa4Zn1Gd1 and (**c**,**d**) MgCa2Zn1Gd3 alloys with selected areas of EDS analyses.

Zinc oxide (ZnO) thin films were deposited on the MgCa4Zn1Gd1 and MgCa2Zn1Gd3 alloys by thermal atomic layer deposition for 500, 1000 and 1500 cycles. The composition of both Mg alloys was investigated in the work of the authors, presented in the article [12]. The SEM images and corresponding EDS analyses of the MgCa4Zn1Gd1 and MgCa2Zn1Gd3 alloys are presented in Figure 1. The cast Mg alloys exhibit dendritic microstructures with solute-rich regions between dendrites (Figure 1a,c). Examination of the alloys’ microstructure studied by SEM-EDS revealed a primary α-Mg phase and eutectics, including (α-Mg + Mg_2_Ca), (α-Mg + Mg_2_Ca + Ca_2_Mg_6_Zn_3_), and (α-Mg + Mg_26_Zn_59_Gd_7_), which are distributed along grain boundaries [12]. It can be observed that the volume of the eutectic decreases with the addition of gadolinium. This is due to the small volume of the intermetallic phases, Mg_2_Ca and Ca_2_Mg_6_Zn_3_, which fill the larger space between the grain boundaries. Furthermore, the Gd-containing phases distributed along the interdendritic regions appear as bright areas [12].

**Figure 2:**
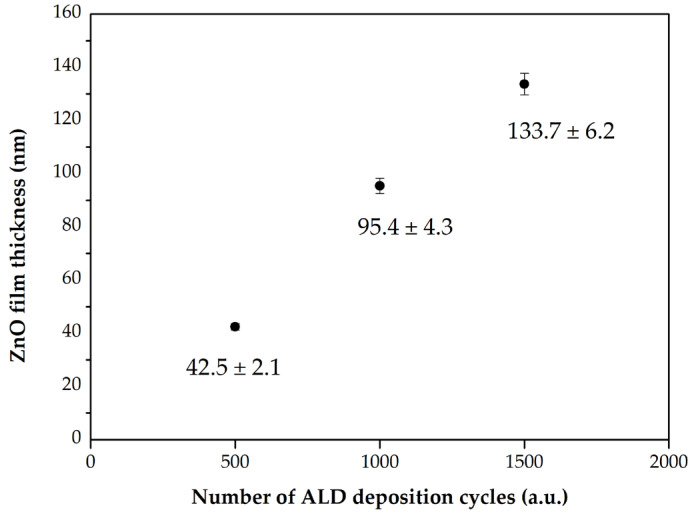
Correlation between the number of ALD cycles and the resulting thickness of the ZnO thin film with the calculated standard deviation (SD) for each layer thickness.

The thickness of the resulting films was characterized using an optical reflectometer. As shown in Figure 2, the film thickness increased linearly with the number of ALD cycles, yielding values of approximately 42.5 ± 2.1 nm, 95.4 ± 4.3 nm, and 133.7 ± 6.2 nm for 500, 1000, and 1500 cycles, respectively. This linear growth is characteristic of a self-limiting ALD process and confirms the controlled deposition of the ZnO coatings.

This linear increase in film thickness indicates a consistent and uniform growth per cycle (GPC), which is characteristic of a self-limiting ALD process. The calculated average GPC for ZnO was determined to be in the range of 0.085–0.095 nm/cycle. These values are in good agreement with those reported in the literature for thermal ALD processes using similar precursors and deposition conditions [13,14]. The slight variation in GPC may be attributed to factors such as surface saturation dynamics, precursor reactivity, and possible changes in nucleation behavior over successive cycles.

**Figure 3:**
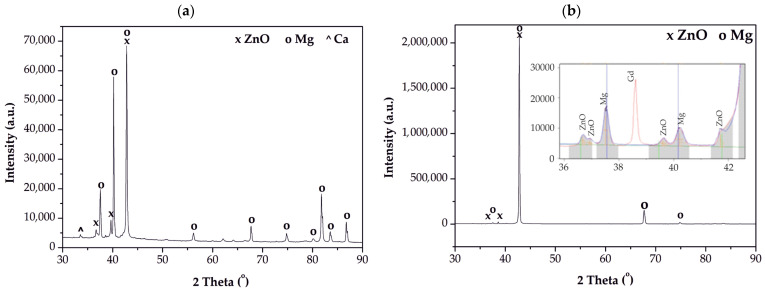
X-ray diffraction patterns of the ZnO film, applied after 1500 cycles to: (**a**) MgCa4Zn1Gd1, and (**b**) MgCa2Zn1Gd3 alloys.

The crystalline structure of the coatings was confirmed by X-ray diffraction (XRD). The diffraction patterns for the coated MgCa4Zn1Gd1 and MgCa2Zn1Gd3 alloys (Figure 3) confirm the presence of a crystalline ZnO phase. The patterns show characteristic peaks for ZnO, which can be indexed to the hexagonal wurtzite structure (space group P6_3_ mc; space group no. 186; lattice parameters: a = 3.2900 Å, b = 3.2900 Å, c = 5.3000 Å according to JCPDS No 98-018-4674). Prominent peaks were observed at 2θ angles of approximately 36.59°, 39.45° and 41.76°, corresponding to the (010), (002), and (011) crystal planes, respectively. In addition to the ZnO peaks, the patterns also contain diffraction peaks from the underlying Mg-based substrate, specifically from Mg (JCPDS No 98-005-2260; crystal system: hexagonal; space group: P 63/m m c), Ca (JCPDS No 96-900-8531; crystal system: cubic; space group: I m −3 m), and Gd (JCPDS No 98-063-5708; crystal system: hexagonal; space group: P 63/m m c) phases, which are primary constituents of the studied alloys.

The average crystallite size (D), determined using the Scherrer equation, was estimated to be approximately 50 nm for the ZnO layer on MgCa2Zn1Gd3 substrate and 70 nm for the ZnO layer on MgCa4Zn1Gd1. It was also found that the growth of ZnO crystals in the layer occurred along the direction perpendicular to the (200) plane. Cuadra et al. [15] also measured the crystallite size using the Scherrer formula for the ZnO thin film applied on glass. The average particle size for this film was estimated to be 25.0 nm, based on the diffraction peak at 2θ = 34.4° that was associated with the reflection plane [15]. The measured crystallite sizes should correspond to the surface topography of the analyzed layers using AFM. The ZnO layer deposited on the MgCa2Zn1Gd3 alloy after 1500 cycles should have a slightly smoother surface compared to the same layer deposited on the other tested alloy [15]. Lower roughness should result in better corrosion protection. Babić et al. [16] found that the small grain structure has a much higher surface-to-volume ratio. It also has a high proportion of inter-grain spaces. This leads directly to improved physical properties, which are crucial for a variety of applications.

**Figure 4:**
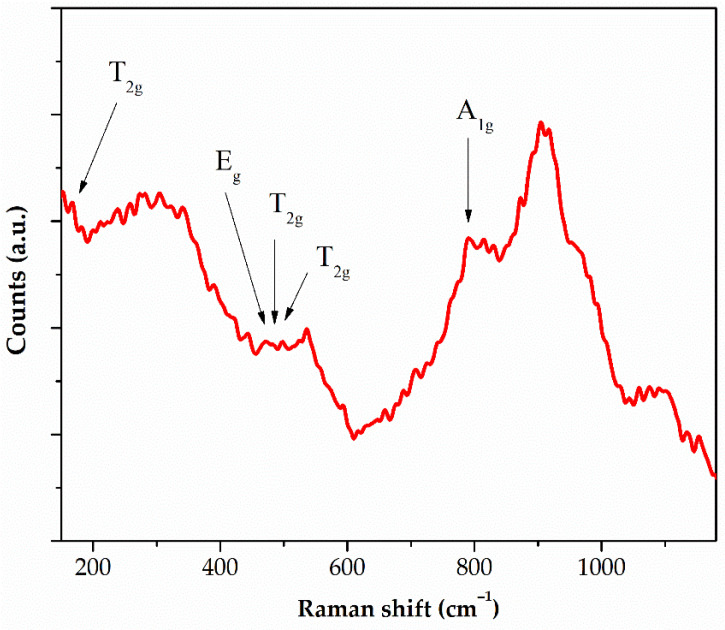
Raman spectrum of the ZnO layer deposited on the MgCa2Zn1Gd3 alloy.

The chemical structure and phase composition of the ZnO layers were further confirmed by Raman spectroscopy. The Raman spectra for the ZnO-coated MgCaZnGd alloys exhibited a high degree of similarity. Figure 4 shows a representative spectrum for the MgCa2Zn1Gd3 alloy with a deposited ZnO layer after 1500 cycles.

The Raman spectrum of the ZnO layer on the MgCa2Zn1Gd3 alloy exhibits a characteristic and intense band at ~437 cm^−1^, which is assigned to the E_2_ (high) phonon mode of crystalline ZnO with a wurtzite structure. This well-defined feature is a clear fingerprint of the ZnO deposited layer. Weaker bands are also observed at approximately ~330 cm^−1^, 380 cm^−1^, and 580 cm^−1^, which are consistent with second-order Raman scattering and can be attributed to defects or lattice imperfections, respectively.

The sharp and well-resolved nature of the dominant Raman bands confirms the successful deposition of a crystalline zinc oxide layer onto alloy surface. The spectral quality indicates good crystallinity of the deposited ZnO film.

**Figure 5:**
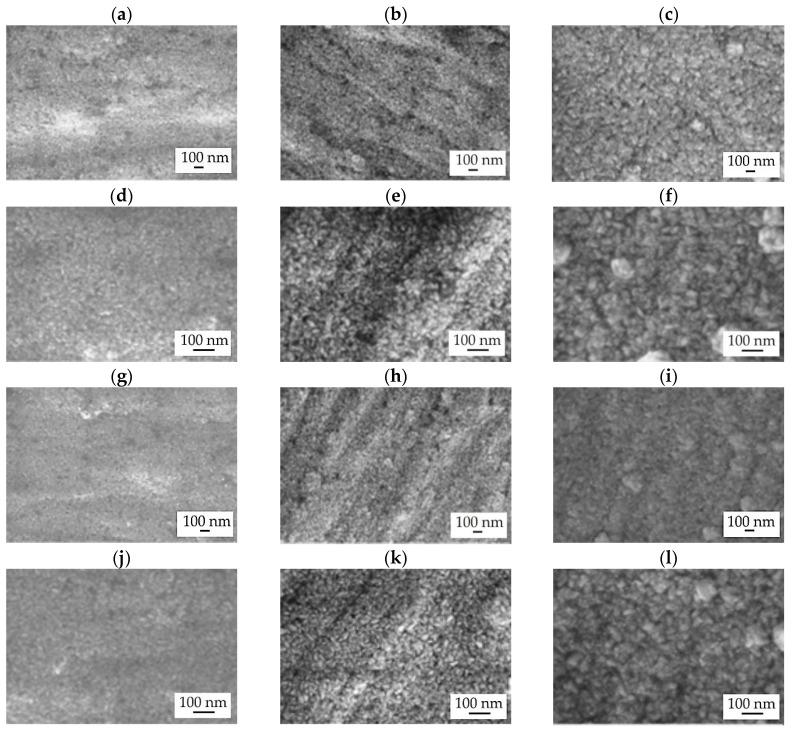
SEM images of ZnO thin films deposited on MgCa4Zn1Gd1 alloy after: (**a**,**d**) 500; (**b**,**e**) 1000; and (**c**,**f**) 1500 cycles, and deposited on MgCa2Zn1Gd3 alloy after: (**g**,**j**) 500; (**h**,**k**) 1000; and (**i**,**l**) 1500 cycles.

The surface morphologies of thin zinc oxides deposited on MgCa4Zn1Gd1 and MgCa2Zn1Gd3 were found to be very similar, as evidenced by SEM observations (Figure 5). It has been observed that the ZnO layers exhibited a homogeneous, granular structure [17], with particles exhibiting an elongated shape [17,18]. Furthermore, an increase in deposition time resulted in larger particles, leading to the formation of agglomerates [18,19] (Figure 5c,f,i,l). Pores were also observed on the surface of the samples, the size of which decreased with the growth of particles and agglomerates. Notably, the ZnO layer on the MgCa2Zn1Gd3 alloy (with 3 wt.% Gd) appeared significantly more compact than that on the MgCa4Zn1Gd1 alloy (with 1 wt.% Gd). This enhanced compactness is directly attributed to the higher gadolinium content. Gadolinium is known to refine the grain structure of the underlying magnesium alloy substrate [20,21]. A finer and more uniform substrate grain structure provides a more homogeneous nucleation site for the growing ZnO layer, promoting the development of a denser, less porous coating. Furthermore, the addition of gadolinium reduces the overall porosity of the Mg-based alloy itself [20] and enhances the formation of a more stable and protective native passive layer [22], which collectively contribute to the improved morphology and expected properties of the deposited ZnO coating. The enhanced compactness and improved morphology of the zinc oxide (ZnO) film on gadolinium-containing magnesium alloys significantly impact the coating’s performance. These characteristics are likely to result in better adhesion between the coating and the substrate, potentially increasing the coated material’s durability and longevity. Furthermore, the denser structure of the ZnO layer could enhance the coating system’s barrier properties, thereby improving its corrosion resistance and overall protective capabilities.

**Figure 6:**
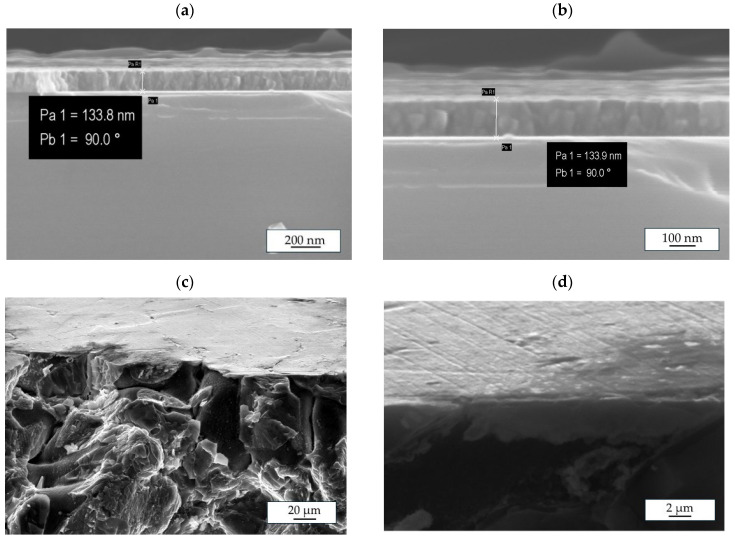
SEM cross-sectional images of the ZnO films deposited after 1500 cycles on: (**a**,**b**) glass, (**c**) MgCa4Zn1Gd1, and (**d**) MgCa2Zn1Gd3 alloys showing the measured film thickness.

SEM micrographs showing the coating cross-section of the ZnO-ALD thin films deposited after 1500 cycles on the glass and MgCa4Zn1Gd1, and MgCa2Zn1Gd3 alloys, with appropriate scale bars, are presented in Figure 6. The measured film thickness corresponds to the ZnO thickness measured with an optical reflectometer (Figure 6a,b). It can be seen that the ZnO coating has a columnar structure, adheres well to the substrate (Mg alloys), and does not delaminate (Figure 6c,d).

**Figure 7:**
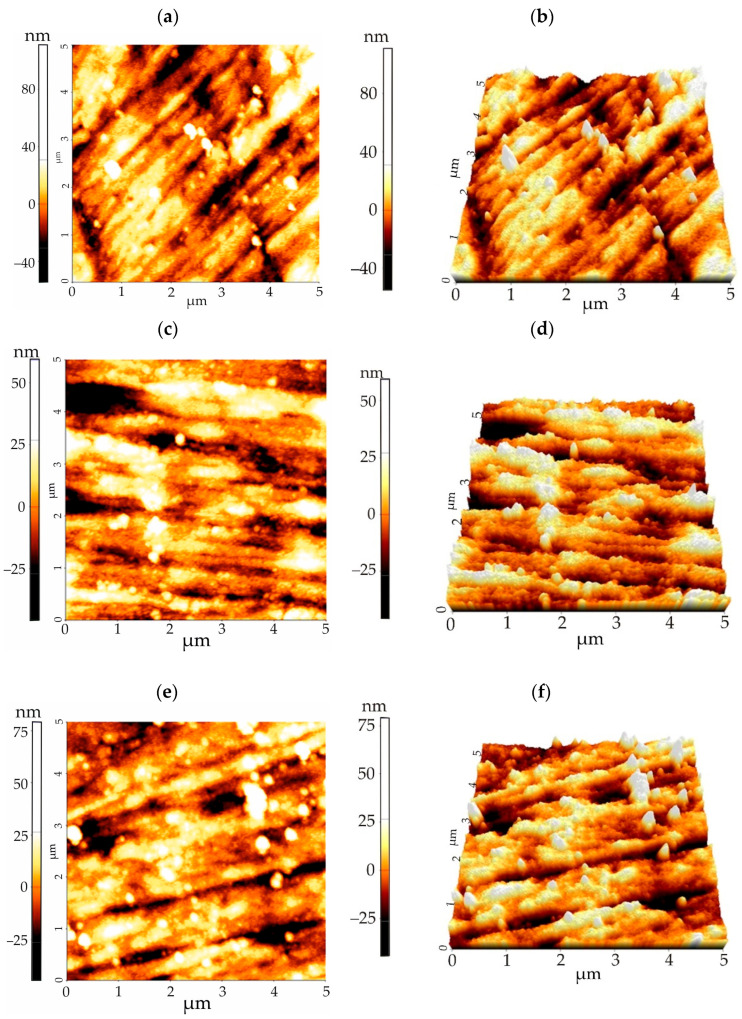

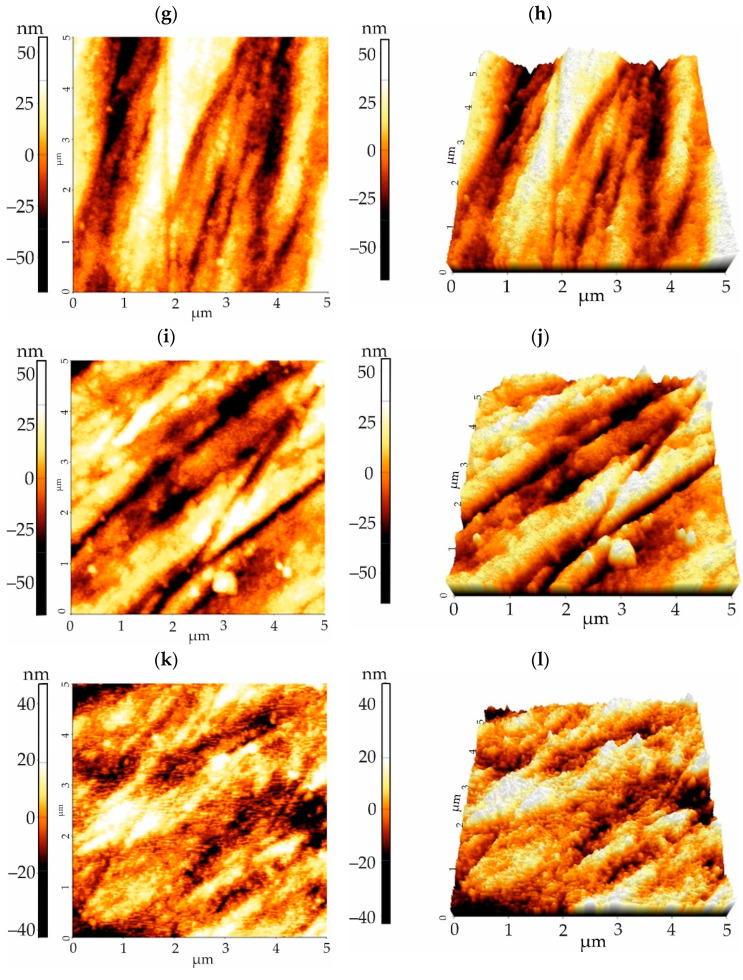
2D and 3D AFM images of ZnO thin films surface topography deposited on MgCa4Zn1Gd1 alloy after: (**a**,**b**) 500 cycles; (**c**,**d**) 1000 cycles; (**e**,**f**) 1500 cycles, and MgCa2Zn1Gd3 alloy after: (**g**,**h**) 500 cycles; (**i**,**j**) 1000 cycles; (**k**,**l**) 1500 cycles.

The surface topography was observed using an atomic force microscope (AFM) to supplement the SEM observations. The analysis of the topography of the thin ZnO films was performed on a 5 µm × 5 µm area. The AFM results (Figure 7) revealed that the ZnO layers exhibited a granular morphology, consistent with the SEM data. Furthermore, the surface roughness of the ZnO layers was found to be influenced by both the underlying alloy substrate and the deposition conditions. Specifically, the increase in surface roughness can be attributed to two primary factors. First, residual thermal strain, which characteristically increases with growth temperature. Second, microstructural strain, which is influenced by the specific environmental conditions during deposition [22]. All samples exhibited homogeneous surfaces. The ZnO films exhibited a comparable granular topography for both magnesium alloys. A key observation was that an increase in ZnO layer thickness correlated with an increase in particle size. The summary of the roughness parameters, including the average roughness R_a_ and the root mean square roughness R_S_, of the ZnO layers is provided in Table 1.

The surface roughness of the ZnO layers was found to be dependent on both their thickness and the substrate alloy composition. A clear trend emerged where thicker layers (deposited after 1500 cycles) resulted in significantly lower roughness. For instance, the 1500-cycle ZnO layer on the MgCa2Zn1Gd3 alloy exhibited the lowest values (R_a_ = 7.65 nm, R_s_ = 9.80 nm), compared to the same layer on the MgCa4Zn1Gd1 alloy (R_a_ = 10.4 nm, R_s_ = 13.49 nm). This confirms that the alloy with 3 wt.% gadolinium provides a superior substrate for growing smoother, thicker ZnO coatings, which is consistent with the measured crystallite size. Conversely, thinner layers (deposited after 500 cycles) displayed elevated roughness, with the highest values observed on the MgCa2Zn1Gd3 alloy (R_a_ = 15.09 nm, R_s_ = 18.52 nm). This trend is consistent with the microscopic observations (Figure 5), which revealed that thicker ZnO films were more compact and homogeneous. An intermediate behavior was noted for the 1000-cycle layers, where the coating on the MgCa2Zn1Gd3 substrate exhibited slightly higher roughness than that on the MgCa4Zn1Gd1 alloy, suggesting a transition in the growth mechanism.

Corrosion is one of the primary processes that causes the degradation of metal implants in the human body. It is known that the corrosion resistance of the studied Mg alloys strongly depends on many factors, including their chemical composition, microstructure, oxide film, and corrosion product film, as well as the composition of the corrosion medium, in this case Ringer solution. It is also known that ZnO layers can be passivated in a chloride environment through the introduction of chloride ions that serve to fill oxygen vacancies, thereby reducing layer defects. However, elevated chloride concentrations can also induce a transformation of ZnO into zinc chloride (ZnCl_2_) and an increase in defects. The effective process of chloride passivation requires strict regulation of environmental conditions, frequently using chloride salts, to achieve the intended results [23]. In the work [24], An et al. investigated the corrosion behavior of ZnO nanosheets in chloride solutions. The results demonstrated that in a solution of NaCl, chloride ions react with ZnO to form ZnCl_2_. At low concentrations of NaCl (approximately 1 wt.%), the ZnO structure demonstrates notable passivation properties. Higher concentrations of chloride ions have been shown to accelerate the transformation of ZnO to ZnCl_2_, thus weakening the passivation properties. As the sodium chloride (NaCl) concentration approaches 3 wt.%, the passivation capability attributable to oxygen vacancies is known to disappear [24].

**Figure 8:**
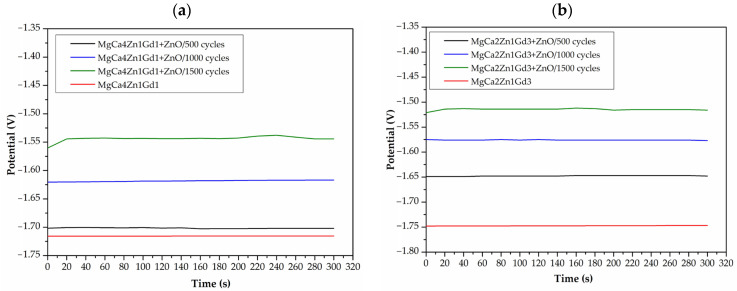
The E_OCP_ changes as a function of immersion time for studied ZnO thin films applied to: (**a**) MgCa4Zn1Gd1; (**b**) MgCa2Zn1Gd3 alloys in Ringer solution at 37 °C.

The protective properties of ZnO thin films deposited on MgCa4Zn1Gd1 and MgCa2Zn1Gd3 alloys were evaluated using open-circuit potential (E_OCP_) measurements. The E_OCP_ curves indicated that the ZnO layers deposited on the Mg alloys are characterized by small fluctuations in a chloride-rich environment (Figure 8). A close examination reveals that the curves are quite similar. In addition, an increase in coating thickness has been observed to correspond with an increase in E_OCP_ values (for ZnO films deposited on the MgCa4Zn1Gd1 alloy after 500, 1000 and 1500 cycles, the E_OCP_ values were −1.702, −1.617 and −1.544 V, respectively, for the same ZnO films desposited on the MgCa2Zn1Gd3, the E_OCP_ values were −1.647, −1.576 and −1.515 V, respectively). This suggests a potential correlation between coating thickness and corrosion resistance, with thicker coatings potentially offering enhanced protection against corrosion. The variations in E_OCP_ among ZnO layers deposited on both Mg alloys are attributable to changes in the alloys’ chemical composition (the difference between Ca and Gd content). Furthermore, the E_OCP_ values were −1.715 and −1.747 V for the alloys with gadolinium additions of 1 and 3 wt.%, respectively.

**Figure 9:**
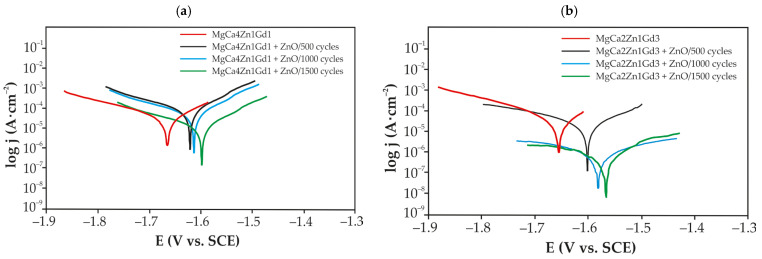
Polarization curves for the ZnO thin films and uncoated Mg-based alloys in Ringer solution at 37 °C: (**a**) MgCa4Zn1Gd1 alloy; (**b**) MgCa2Zn1Gd3 alloy.

Electrochemical tests of ZnO thin films and uncoated alloys were performed in Ringer solution at 37 °C (Figure 9). It has been observed that the shapes of all Tafel curves are similar to each other. Furthermore, an increase in the thickness of the ZnO layer on Mg alloys has been shown to correspond with an increase in the corrosion potential. In addition, the substrate magnesium alloys exhibit a similar potential value. The corrosion current values of the ZnO layers deposited on different Mg alloys exhibit variability, as evidenced by the variation in the curves. In the case of ZnO-ALD deposited on MgCa4Zn1Gd1, the Tafel curves are located at higher j_corr_ values (10^−4^–10^−3^ A·cm^−2^) compared to ZnO films deposited on MgCa2Zn1Gd3 (10^−6^–10^−4^ A·cm^−2^).

The primary electrochemical parameters resulting from the Tafel extrapolation of the polarization curves are presented in Table 2. The theoretical framework suggests a direct correlation between a higher corrosion potential value and a lower corrosion current density value, indicating increased corrosion resistance [25]. The results indicate that the ZnO layer deposited after 1500 cycles on the MgCa2Zn1Gd3 alloy exhibits a reduced corrosion current and an increase in polarization resistance value (j_corr_ = 0.7 × 10^−3^ mA·cm^−2^, R_p_ = 59,480 Ω·cm^2^) compared to the ZnO layer deposited on the second alloy tested (j_corr_ = 29.2 × 10^−3^ mA·cm^−2^, R_p_ = 1800 Ω·cm^2^). Additionally, the corrosion properties of the ZnO layers deposited on the Mg alloy with 3 wt.% gadolinium were found to be superior to those of the ZnO layers deposited on the Mg alloy with 1 wt.% Gd.

Furthermore, it was observed that Mg alloy with higher Gd content had slightly lower j_corr_ value compared to the Mg alloy with lower Gd content (58.6 × 10^−3^ and 41 × 10^−3^ mA·cm^−2^ for MgCa4Zn1Gd1 and MgCa2Zn1Gd3 alloys, respectively) and very similar R_p_ (900 and 903 Ω·cm^2^ for MgCa4Zn1Gd1 and MgCa2Zn1Gd3, respectively). The findings of this study also suggest that ZnO-ALD after 1500 cycles on the MgCa2Zn1Gd3 alloy offers enhanced corrosion protection within a chloride-rich environment (Table 2). Zeng et al. [26] obtained higher values of corrosion potential and corrosion current density than in the present study. These researchers investigated the corrosion mechanism of cast and extruded Mg–1.21Li–1.12Ca–1Y alloys. The corrosion potential of extruded Mg–1.21Li–1.12Ca–1Y and cast alloys was −1.67 and −1.69 V, respectively. The corrosion current density was determined to be 6.67 × 10^−5^ and 8.44 × 10^−5^ A·cm^−2^ for the extruded and cast alloys, respectively. The work [27] presented the corrosion behavior of a thin zinc oxide (ZnO) coating deposited on a magnesium alloy (Mg-Zn-Zr) by electrophoretic deposition. The corrosive environment was Ringer solution. The ZnO coating was found to have improved corrosion resistance, as confirmed by an electrochemical test. Preliminary findings from a polarization test indicated an order of magnitude decrease in current density compared to the baseline of the uncoated alloy.

Additionally, the corrosion rates (CR) were calculated from potentiodynamic studies. The CR (mm/y) was calculated according to Formula (1) [28,29]:

(1) $$\mathrm{CR}\;(\mathrm{mm}/y)=3.28\;\times \;\frac{M\cdot I}{n\cdot d}$$

where M—atomic mass (u), I—corrosion current density (mA·cm**^−^**^2^), n—number of electrons involved in the corrosion reaction, d—density (g·cm**^−^**^3^).

The corrosion rate (CR) for ZnO thin films deposited after 1500 cycles on the Mg alloys was 0.65 and 0.016 mm/y for the MgCa4Zn1Gd1 and MgCa2Zn1Gd3 alloys, respectively. The corrosion rates for the ZnO films applied to MgCa4Zn1Gd1 were 1.15 and 1.01 mm/y for 500 and 1000 deposition cycles, respectively. Lower CR values were obtained for the same films deposited on the second tested alloy, measuring 0.54 and 0.026 mm/y for 500 and 1000 deposition cycles, respectively. These results are consistent with those reported in the literature [28,29]. Roh et al. discussed the influence of 0.1 and 0.3 wt.% Ca on the anticorrosion properties of the Mg-Zn alloy in Hank’s solution. Polarization tests determined corrosion rates of 0.11 and 0.39 mm/y for Ca contents of 0.1 and 0.3 wt.%, respectively. The use of tannic acid to obtain natural coatings on two different magnesium alloys, AZ31 and AZ91, for biomedical applications is presented in the paper [29]. Corrosion tests were conducted at room temperature in phosphate-buffered saline (PBS), Ringer and Hank solutions. The CR in Ringer solution for the AZ31TA_20 and AZ91TA coatings (0.04 and 0.18 mm/y, respectively) was slightly lower than that of the 1500 Zn-ALD film applied to MgCa2Zn1Gd3 [29].

**Figure 10:**
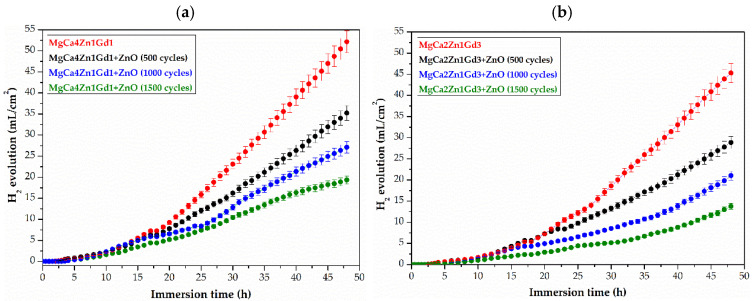
Hydrogen evolution volume as a function of immersion time in Ringer solution at 37 °C for 48 h for the ZnO thin films applied to: (**a**) MgCa4Zn1Gd1, and (**b**) MgCa2Zn1Gd3 alloys and uncoated alloys.

An in vitro immersion test in body fluids has been shown to be a reliable predictor of the corrosion behavior of Mg-based alloys in vivo. Prolonged immersion test is regarded as offering a more realistic simulation of actual usage conditions than accelerated electrochemical testing methods. In order to enhance the corrosion resistance of Mg alloys, surface modification is a significant method of advancement. The corrosion behavior of Mg-based alloys coated with ZnO thin films was observed during 48 h of immersion tests in Ringer solution at a temperature of 37 °C (Figure 10). Corrosion resistance was evaluated by measuring the volume of evolved hydrogen gas (H_2_), a direct byproduct of the anodic corrosion reaction of magnesium. This technique has proven to be a highly effective means of estimating the amount of hydrogen evolving during the implantation period, which helps to determine the resulting hemolysis in cells [30].

The H_2_ evolution curves for all tested samples of the MgCa4Zn1Gd1 and MgCa2Zn1Gd3 alloys exhibited similar kinetics. The highest volume of released H_2_ was obtained for ZnO layers deposited on the MgCa4Zn1Gd1 alloy. The H_2_ results obtained after 48 h of immersion in the corrosive environment were 35.23, 27.11, and 19.34 mL·cm^−2^ after 500, 1000 and 1500 deposition cycles, respectively. In contrast, the ZnO layers deposited on the MgCa2Zn1Gd3 alloy (with 3 wt.% Gd) resulted in reduced levels of hydrogen gas released, confirming the slower degradation rate. For a zinc oxide layer deposited after 1500 cycles, the hydrogen volume was measured to be 13.77 mL·cm^−2^. This trend is consistent with the results of the roughness measurements, where the ZnO layer on the MgCa2Zn1Gd3 alloy after 1500 cycles exhibited lower roughness values (R_a_ = 7.65 nm, R_s_ = 9.80 nm) compared to the layer on the MgCa4Zn1Gd1 alloy (R_a_ = 10.4 nm, R_s_ = 13.49 nm). This correlation suggests that the denser and more compact coating (as indicated by the lower roughness) provides a superior barrier, resulting in less hydrogen evolution. The hydrogen volumes for the MgCa2Zn1Gd3 alloy with ZnO layers from 1000 and 500 cycles were 21.03 and 28.87 mL·cm^−2^, respectively. However, the volume of hydrogen released is related not only to the roughness parameters but also to the type of substrate on which the ZnO layers were deposited. In our study, the amount of gadolinium and calcium in the studied alloys, as well as the high-chloride corrosion environment, also plays an important role. As mentioned above, the presence of gadolinium in the Mg alloy improves corrosion resistance. Calcium has been demonstrated to enhance corrosion protection through suppression of microgalvanic couples, its action on grain structure refinement, leading to the formation of a more protective surface film [31]. However, a higher concentration of Ca in the Mg alloy (4 wt.% for the alloy with 1 wt.% Gd) in a chloride environment can accelerate corrosion because Ca then increases the conductivity of the electrolyte [32]. Furthermore, the uncoated MgCa2Zn1Gd3 alloy itself released less hydrogen (45.30 mL·cm^−2^) than the uncoated MgCa4Zn1Gd1 alloy (52.13 mL·cm^−2^). These results collectively demonstrate that the alloy with 3 wt.% gadolinium, both in coated and uncoated states, possesses superior corrosion resistance. That is because gadolinium is characterized by high solubility in Mg solid solution at eutectic temperatures. The distribution of this element in the Mg matrix serves to reduce the corrosion rate of magnesium alloys. This phenomenon is associated with the formation of the crystallographic β phase, which is resistant to corrosion [33]. However, a minor addition of calcium (for example, 2 wt.%) to the alloy has been shown to improve the overall pitting corrosion resistance of the material to pit and reduce the corrosion current density of the magnesium alloy [34]. In the work [34], Istrate et al. investigated the corrosion resistance of Mg0.5CaZn alloys (with 0.5, 1.5, and 3 wt.% Zn addition) in SBF solution. The corrosion rate was found to be comparable for all alloys (7.96, 8.11, 7.03 mm/y for Mg0.5Ca0.5Zn, Mg0.5Ca1.5Zn, Mg0.5Ca3.0Zn, respectively) with a marginally lower rate observed for the 3 wt.% Zn alloy. The basic corrosion parameters obtained from the Tafel analysis were the corrosion potential, E_corr_, the density of the corrosion current, j_corr_ and the polarization resistance, R_p_, which for this alloy were E_corr_ = 1.458 V, j_corr_ = 32.26 µA·cm^−2^ and R_p_ = 1.47 kΩ·cm^2^. This phenomenon can be attributed to the passivating effect of zinc oxide on the corrosion resistance of the material studied.

For context, Rajan et al. [35] investigated the anticorrosion properties of an Mg–Al_6_–Zn_1.5_-Cu_2_-Ge_0.5_ alloy by modifying its surface with nanocomposite layers of Ta_2_O_5_ and Nb_2_O_5_. The hydrogen emission from both uncoated and coated alloys was assessed over a period of 120 h by immersion in a synthetic body fluid (SBF). At the point of initial immersion, the hydrogen evolution rate was found to be significantly higher for the alloy than for the coated substrates. The low electrochemical potential of the Mg alloy results in the formation of hydrogen bubbles upon immersion in the electrolyte, regardless of any coating. As the duration of the immersion increases, the degradation rate accelerates, and pits begin to form on the Mg alloy. However, the alloys coated with Ta_2_O_5_ and Nb_2_O_5_ were effectively isolated, preventing the penetration of SBF and thus mitigating corrosion. This parallels the protective role observed in our study for the ZnO coating, particularly on the Gd-rich substrate. It is worth mentioning here that gadolinium has a greater affinity for oxygen compared to magnesium. Therefore, its corresponding corrosion products, such as GdO_3_ and MgGd_2_O_4_, appear on the alloy surface and act as an effective diffusion barrier, preventing further oxidation [36].

**Figure 11:**
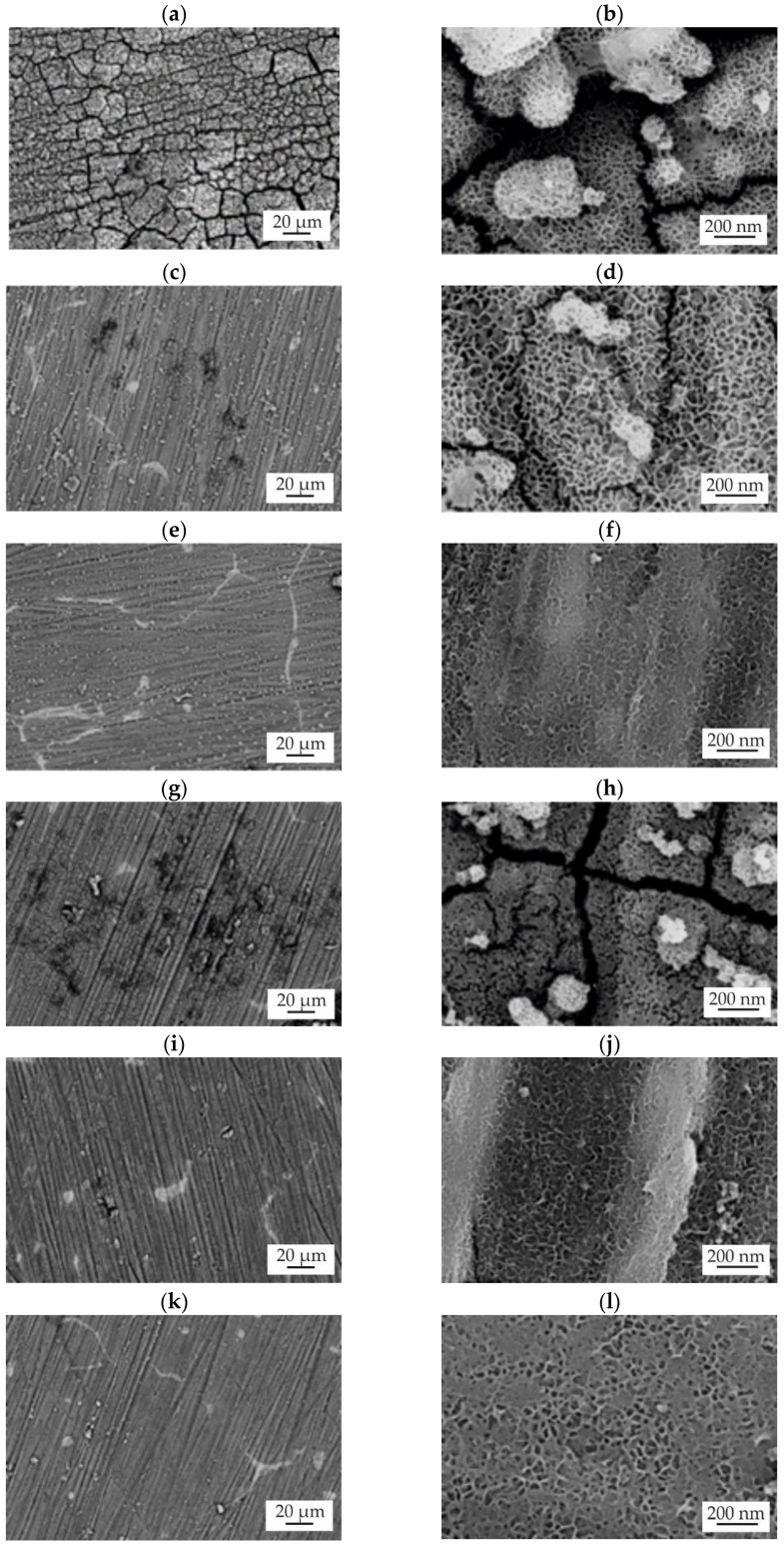
SEM images of the samples’ surfaces with corrosion products of the ZnO films deposited onto MgCa4Zn1Gd1 alloy after: 500 cycles (**a**,**b**), 1000 cycles (**c**,**d**), 1500 cycles (**e**,**f**), and applied to MgCa2Zn1Gd3 alloy after: 500 cycles (**g**,**h**), 1000 cycles (**i**,**j**), and 1500 cycles (**k**,**l**) after 48 h of immersion in Ringer solution at 37 °C.

Subsequent to an immersion test period of 48 h, a SEM examination of the sample surfaces was performed (Figure 11). The surfaces exhibited a network of cracks, which are likely artifacts from dehydration during the sample drying process. Critically, all ZnO films also showed evidence of perforations, which are indicative of localized pitting corrosion [37]. These perforations serve as direct pathways for the entry of corrosive solution to the underlying substrate, thereby accelerating the degradation process [38]. A key observation was that the ZnO coating deposited after 1500 cycles in both alloys demonstrated smaller and fewer microcracks (Figure 11e,k), suggesting improved structural integrity and cohesion. Furthermore, the grain boundaries of the underlying alloy were visible in most samples, with the exception of the ZnO layer deposited on MgCa4Zn1Gd1 after 500 cycles. In all cases, the surface was covered with corrosion products that exhibited a porous, spongy morphology. In particular, this layer of corrosion products was denser and more continuous in the samples with ZnO coatings deposited after 1500 cycles, particularly on the MgCa2Zn1Gd3 alloy. The observed detachment of some corrosion products from the ZnO surfaces underscores the dynamic and unstable nature of the corrosion layer, which can lead to fluctuating corrosion rates and the release of particles into the surrounding environment.

**Figure 12:**
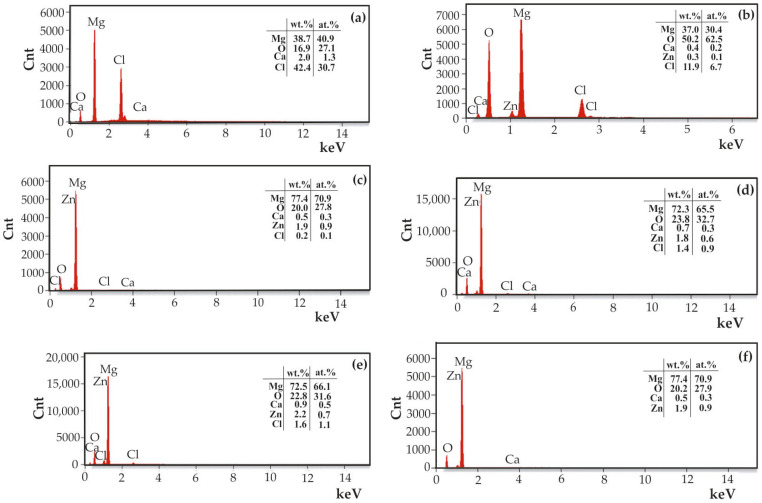
EDS analysis of corrosion products of the ZnO thin films applied to MgCa4Zn1Gd1 alloy after: (**a**) 500 cycles, (**b**) 1000 cycles, (**c**) 1500 cycles, and MgCa2Zn1Gd3 alloy after: (**d**) 500 cycles, (**e**) 1000 cycles, (**f**) 1500 cycles.

Microscopic observations were supplemented with an EDS analysis of corrosion products (Figure 12). The results demonstrated that the corrosion products comprised Mg, O, Zn, Ca, and Cl for ZnO samples deposited after 500 and 1000 cycles in alloys of MgCa4Zn1Gd1 and MgCa2Zn1Gd3, respectively. Following a 48 h immersion in Ringer’s solution, the ZnO layer deposited after 500 cycles on the MgCa4Zn1Gd1 underwent complete dissolution. No reflections from Zn were identified, likely due to the extended corrosion test duration for such a thin layer. A uniformly applied film provides initial corrosion protection. With the progression of time, the chloride ions present in the Ringer solution disrupt the nanometer-thin protective film, leading to chemical reactions that generate corrosion products. These corrosion products, in turn, play a crucial role in determining the corrosion resistance of the material. In this study, the ZnO layers were found to be particularly thin and porous. The presence of nanopores within the ZnO film has been demonstrated to be an inadequate form of corrosion protection. These nanopores have been shown to permit the penetration of chloride ions, resulting in the occurrence of corrosion at the interface between the thin film and the substrate. This corrosion process has been observed to involve the dissolution of the protective layers.

Additionally, no chlorine was found in the corrosion products of ZnO samples deposited after 1500 cycles (for the MgCa2Zn1Gd3 alloy), or only a small amount was found (0.2 wt.% Cl for the MgCa4Zn1Gd1 alloy). The primary elements detected in these samples were Mg, O, Ca, and Zn. This finding indicates that magnesium and oxygen may potentially originate from Mg(OH)_2_, which is the primary and predominant corrosion product of magnesium alloys. After the sample is immersed in the simulated body fluid, anodic dissolution of magnesium occurs, leading to the formation of a magnesium hydroxide layer on the sample surface. The chloride ions of the Ringer solution (with a concentration of approximately 0.15 M) tend to accumulate in regions where the Mg(OH)_2_ layer is thinner and porous, resulting in the formation of soluble magnesium chloride [26,36,39,40]. As a result, Mg^2+^ and Zn^2+^ ions are released into the solution. With prolonged immersion time, the concentration of Zn^2+^ ions increases due to the ongoing dissolution of zinc. Subsequently, Zn(OH)_2_ begins to precipitate selectively, providing a protective barrier against corrosion. This increases the protective properties of the corrosion film because it is more stable and insoluble in aqueous solution [41,42].

The protective layer of Mg(OH)_2_ formed on magnesium-based alloys is strongly dependent on environmental parameters such as pH and chloride ion concentration. Because of this, in chloride-rich or aggressive environments, a secondary protection mechanism—such as formation of a Zn-rich hydroxide/oxide layer (e.g., Zn(OH)_2_) on a studied Mg-based—may indeed be beneficial in enhancing corrosion resistance [37].

**Figure 13:**
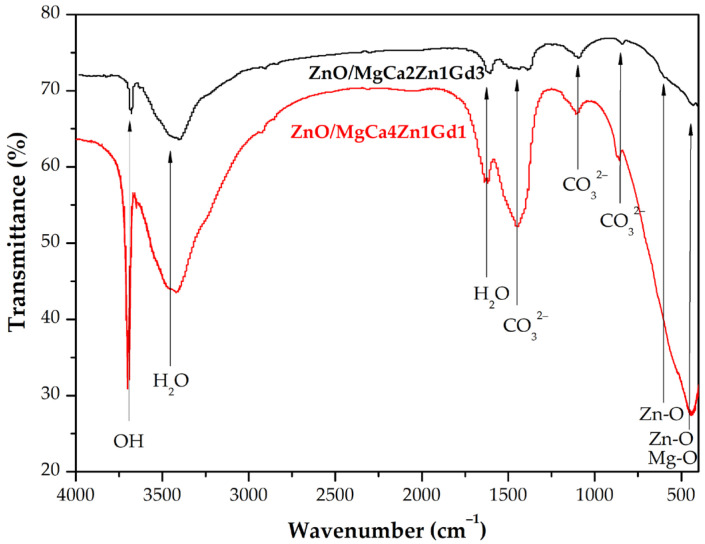
FTIR spectra of corrosion products collected from the surface of MgCa4Zn1Gd1 and MgCa2Zn1Gd3 alloys with deposited ZnO thin films after 48 h of immersion in Ringer solution at 37 °C.

Following the immersion test, the corrosion products were analyzed using FTIR spectroscopy to identify their chemical composition. The results are presented in Figure 13. Given the spectral similarity observed across all samples, the analysis focused on the ZnO coatings deposited after 1500 cycles on both alloys as representative cases. The FTIR spectra for all tested samples confirmed that the primary corrosion products were magnesium hydroxide and magnesium carbonate.

For the studied samples, there are characteristic peaks at 3400 cm^−1^ attributed to O-H stretching vibrations and 1640 cm^−1^ attributed to H-O-H bending vibrations originating from water [43]. Critically, the sharp peaks at approximately 3700 and 3645 cm^−1^ are distinctive for O-H stretching in crystalline magnesium hydroxide (Mg(OH)_2_), confirming its formation [44,45,46]. Furthermore, the spectra confirm the formation of carbonates, as evidenced by a broad and complex band between 850 and 1450 cm^−1^, which is characteristic of the vibration modes (C-O stretching and bending) of the carbonate ion (CO_3_^2−^) in compounds such as magnesium carbonate (MgCO_3_) [47]. Finally, the presence of the coating and oxide compounds is verified by bands in the low-frequency region, demonstrating symmetric stretching vibrations for Zn-O (approximately 450 and 550 cm^−1^) [47,48,49] and Mg-O (approximately 440 cm^−1^) [50,51].

The findings of this investigation collectively demonstrate that Mg(OH)_2_ and MgCO_3_ are dominant corrosion products after 48 h of immersion in Ringer solution, but small amounts of Zn-O and Mg-O are also visible. The corrosion mechanism likely began with the formation of a Mg(OH)_2_ layer, which subsequently reacted with atmospheric CO_2_ dissolved in the electrolyte to form MgCO_3_. This sequence is consistent with the established corrosion pathway for magnesium in aqueous environments. Furthermore, the characteristic lamellar morphology of the corrosion products observed by SEM is typical of crystalline Mg(OH)_2_, thus confirming its identity as the primary phase.

**Figure 14:**
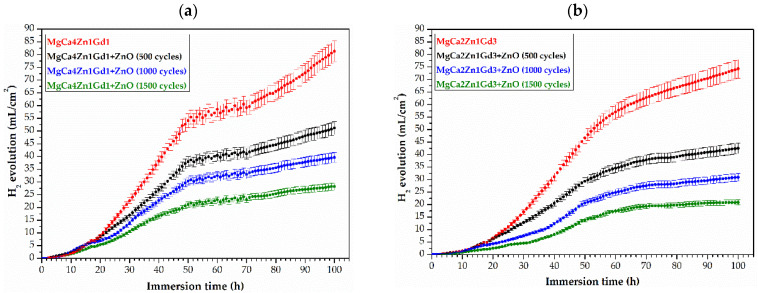
Hydrogen evolution volume as a function of immersion time in Ringer solution at 37 °C for 100 h for the ZnO thin films applied to: (**a**) MgCa4Zn1Gd1, and (**b**) MgCa2Zn1Gd3 alloys and uncoated alloys.

Identification of Mg(OH)_2_ and Zn in the corrosion products after 48 h of immersion in Ringer solution required extending the immersion tests to 100 h (Figure 14). After 48 h, the amount of H_2_ released was comparable to that of previous corrosion tests. For ZnO deposited on the MgCa4Zn1Gd1 alloy, the volume of released H_2_ was 36.11, 28.67, and 19.81 mL·cm^−2^ for layers deposited after 500, 1000, and 1500 cycles, respectively. For ZnO deposited on MgCa2Zn1Gd3, the H_2_ values were 27.72, 19.27, and 12.71 mL·cm^−2^ for ZnO-ALD deposited after 500, 1000, and 1500 cycles, respectively.

After 100 h of immersion, the amounts of hydrogen released for the ZnO layers deposited on MgCa4Zn1Gd1 were as follows: 51.13, 39.66, and 28.26 mL·cm^−2^. Whereas the volumes of released H_2_ for the same thicknesses of ZnO films deposited on the alloy with a higher gadolinium content were: 42.52, 30.92, and 20.89 mL·cm^−2^. For the substrate, the volume of released H_2_ was 81.30 and 74.27 mL·cm^−2^ for the MgCa4Zn1Gd1 and MgCa2Zn1Gd3 alloys, respectively. Observations of the amount of H_2_ released during 100 h of immersion indicate corrosion stabilization for the ZnO layers deposited on the MgCa4Zn1Gd1 alloy between hours 50 and 70 of testing. After this time, an increase in the volume of released H_2_ is visible. A slightly different situation occurred for the ZnO-ALD films deposited on the second alloy tested. In this case, corrosion stabilization continued after 60 h. This may indicate improved corrosion resistance of both the ZnO layers deposited on the MgCa2Zn1Gd3 alloy and the substrate itself.

Furthermore, mass loss was observed and measured during the immersion tests. The mass loss rate results after 100 h of immersion in a corrosive environment, defined as the corrosion rate—CR (mg/cm^2^∙day), were summarized in Table 3. Corrosion rate values are lower for zinc oxide (ZnO) layers deposited on alloy containing greater concentration of gadolinium. The ZnO layer deposited after 500 cycles exhibits a CR index that is half that of the same layer deposited on MgCa4Zn1Gd1. However, it is worth noting that the CR values for ZnO layers deposited after 1500 cycles on both Mg alloys are very similar (0.23 and 0.21 mg/cm^2^·day for ZnO applied to MgCa4Zn1Gd1 and MgCa2Zn1Gd3, respectively). Conversely, the corrosion rate of the MgCa2Zn1Gd3 alloy is significantly lower than that of the other alloy under consideration (2.12 and 1.26 mg/cm^2^·day for MgCa4Zn1Gd1 and MgCa2Zn1Gd3, respectively). This phenomenon may be attributed, in part, to the chemical composition of the alloys.

The corrosion resistance of ultralight Mg-Li alloys was studied by the authors of [52]. The Mg-4%Li, Mg-14%Li, and Mg-7.5%Li alloys were tested in a solution of 0.1 M NaCl. The results showed that the corrosion resistance depends on the chemical composition and crystallographic structure of Mg-Li alloys. Immersion studies indicated that Mg-7.5Li released the highest volume of H_2_ during a 5-day immersion. The Mg-14Li alloy released a small volume of hydrogen. The measured mass loss rates of the Mg-Li alloys allow us to conclude that the average mass loss rates determined for Mg-4Li, Mg-7.5Li, and Mg-14Li over five days were: 0.88, 1.61, and 0.41 mg/cm^2^∙day, respectively. Therefore, the mass loss rate of the Mg-14Li alloy is half that of the Mg-4Li alloy. These results are slightly higher than those obtained in the present study. The corrosion resistance of Mg0.8Ca, ZQ63 (Mg-0.8Ca-5Zn-2.5Ag), and ZQ71 (Mg-0.8Ca-5Zn-1.5Ag) alloys in SBF solution was investigated in [53]. The corrosion rates, measured by the hydrogen release rates for magnesium alloys over 240 h, were 7.58, 4.76, and 2.90 mL/cm^2^∙day for Mg0.8Ca, ZQ63, and ZQ71, respectively. It should be noted that the hydrogen release rate of pure Mg at 37 °C is 40 mL/cm^2^∙day [53].

**Figure 15:**
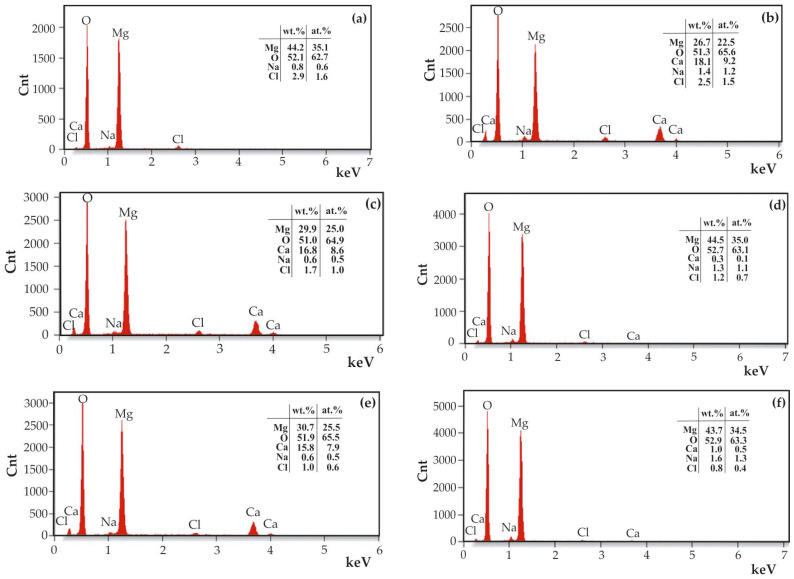
EDS analysis of corrosion products of the ZnO thin films applied to MgCa4Zn1Gd1 alloy after: (**a**) 500 cycles, (**b**) 1000 cycles, (**c**) 1500 cycles, and MgCa2Zn1Gd3 alloy after: (**d**) 500 cycles, (**e**) 1000 cycles, (**f**) 1500 cycles, after 100 h of immersion in Ringer solution at 37 °C.

Following a 100 h immersion in Ringer’s solution, the corrosion products were subjected to further analysis using SEM-EDS (Figure 15). While Zn was not identified in all of the tested samples, Cl was detected. Chlorine concentrations are not significant, with a maximum of 2.9 wt.% detected in the ZnO sample deposited on the MgCa4Zn1Gd1 alloy after 500 cycles and only 0.8 wt.% for the ZnO layer deposited on the MgCa2Zn1Gd3 alloy after 1500 cycles. The most probable origin of the chloride ions in the samples is from the Ringer’s solution, with the chloride ions being present as NaCl and CaCl_2_. The presence of MgCl_2_ is also a possibility, though the high oxygen content in the corrosion products may indicate the presence of Mg(OH)_2_.

**Figure 16:**
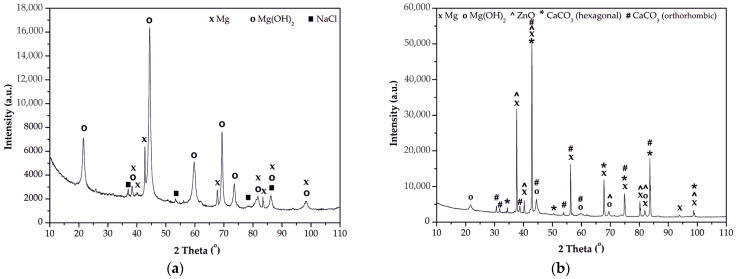
X-ray diffraction patterns of corrosion products for the ZnO film, applied after 1500 cycles to: (**a**) MgCa4Zn1Gd1, and (**b**) MgCa2Zn1Gd3 alloys.

To obtain a more comprehensive evaluation, the EDS results were complemented with phase XRD analysis. It has been demonstrated that, after 100 h of immersion in Ringer’s solution, all ZnO layers deposited on the same alloys exhibited analogous XRD phase analyses. Consequently, the work presents phase analyses of corrosion products for ZnO-ALD films deposited on MgCa4Zn1Gd1 and MgCa2Zn1Gd3 alloys after 1500 cycles (Figure 16).

The compositions of the corrosion products of the zinc oxide (ZnO) layers deposited on magnesium (Mg) alloys differ slightly. For ZnO deposited on the alloy with lower Gd content, the corrosion products are Mg (JCPDS No 98-064-2655; crystal system: hexagonal; space group: P 63/m m c), Mg(OH)_2_ (JCPDS No 98-016-9979; crystal system: hexagonal; space group: P–3 m 1), and NaCl (JCPDS No 98-016-5592; crystal system: cubic; space group: F m–3 m). Phase analysis results confirm SEM-EDS analysis results. The absence of Zn reflections in the sample may indicate a break in the ZnO coating and progressive corrosion, consistent with the results of immersion studies.

The corrosion products of the ZnO layer deposited on the MgCa2Zn1Gd3 alloy included Mg and Mg(OH)_2_, with a significantly higher intensity than the ZnO corrosion products on the other tested alloy. CaCO_3_ was also identified in the corrosion products in two forms: calcite (JCPDS No 98-042-3568; crystal system: hexagonal; space group: R–3 c) and aragonite (JCPDS No 98-005-6090; crystal system: orthorhombic; space group: P n m a). It is important to note that carbonates are less affected by chloride environments than Mg(OH)_2_ and can slightly reduce corrosion rates [54,55]. The presence of Zn ((JCPDS No 98-016-5012; crystal system: hexagonal; space group: P 63 m c) in the corrosion products may indicate the presence of a ZnO coating after 100 h of immersion in Ringer’s solution. The phase analysis results corresponded to the immersion test results, in which stabilization of the corrosion process was observed after 60 h.

## 4. Conclusions

ZnO films offer numerous advantages, including low toxicity, biocompatibility, and biological safety. Consequently, they are optimally suited for biomedical applications. However, the morphology of ZnO nanoparticles plays a significant role in classifying this material for implant applications. The present study investigated the influence of the morphology and topography of ZnO thin films on the corrosion behavior of MgCa4Zn1Gd1 and MgCa2Zn1Gd3. It is evident that the results obtained have enabled the formulation of the following conclusions:

1. X-ray diffraction and Raman spectroscopy confirmed that the thin films deposited on the MgCa4Zn1Gd1 and MgCa2Zn1Gd3 alloys are composed of crystalline ZnO with a wurtzite structure.
2. The ZnO layers exhibited a homogeneous, granular structure composed of elongated particles.
3. The calculated average crystal size is approximately 50 nm for the ZnO layer deposited after 1500 cycles on MgCa2Zn1Gd3 and 70 nm for the same ZnO layer on MgCa4Zn1Gd1 substrate.
4. The surface roughness of the layers was directly correlated with their thickness. ZnO layers deposited after 1500 cycles had the lowest roughness values. Furthermore, the ZnO layer deposited on the MgCa2Zn1Gd3 alloy had lower roughness values, by approximately 30%, compared to those deposited on the MgCa4Zn1Gd1 alloy (the R_a_ value was 7.65 nm and R_s_ was 9.8 nm, compared to R_a_ = 10.4 nm and R_s_ = 13.49 nm), which may suggest improved corrosion resistance.
5. The results of the potentiodynamic tests in Ringer solution demonstrated that the ZnO layer deposited after 1500 cycles on MgCa2Zn1Gd3 exhibited a reduced corrosion current density and an increased polarization resistance value (j_corr_ = 0.7 × 10^−3^ mA·cm^−2^, R_p_ = 59,480 Ω cm^2^) in comparison to the same layer deposited on the second tested alloy (j_corr_ = 29.2 × 10^−3^ mA·cm^−2^, R_p_ = 18,000 Ω cm^2^).
6. The results of immersion studies confirmed the electrochemical results and demonstrated that the MgCa4Zn1Gd1 alloy with ZnO coating exhibited the lowest corrosion resistance after 48 h of testing. The H_2_ volume measurements for these samples were 35.23, 27.11, and 19.34 mL·cm^−2^ after 500, 1000, and 1500 deposition cycles, respectively. In contrast, lower amounts of released hydrogen were obtained for ZnO deposited on the MgCa2Zn1Gd3 alloy. For ZnO layers deposited after 1000 and 500 cycles, the volume of released H_2_ was 21.03 and 28.87 mL·cm^−2^, respectively. The ZnO-ALD coating deposited after 1500 cycles reduced the volume of H_2_ by approximately 70% compared with the uncoated Mg alloy, demonstrating its potential as a protective layer for biodegradable implants.
7. Observations of the amount of released H_2_ during 100 h of immersion indicate anticorrosion stabilization of ZnO layers deposited on the MgCa4Zn1Gd1 alloy between the 50th and 70th hour of testing, followed by an increase in the volume of released H_2_. For ZnO-ALD layers deposited on an alloy with a higher Gd content, corrosion stabilization occurred after the 60th hour of immersion.
8. The corrosion rates (CR) calculated after 100 h of immersion in Ringer solution were very similar for ZnO layers deposited on both Mg alloys after 1500 cycles, and they were 0.23 and 0.21 mg/cm^2^∙day for ZnO applied to MgCa4Zn1Gd1 and MgCa2Zn1Gd3, respectively. The CR calculated from the potentiodynamic tests for the same thin films was 0.65 and 0.016 mm/y for the alloys ZnO deposited on the MgCa4Zn1Gd1 and MgCa2Zn1Gd3 alloys, respectively.
9. SEM observations of ZnO samples after immersion in Ringer solution indicated that, in all cases studied, the corrosion products exhibited a porous and spongy morphology. This morphology was denser for ZnO films deposited after 1500 cycles. Furthermore, the EDS analysis corroborated the composition of the corrosion layer, revealing elements such as Mg, O, Zn, and Ca. The denser morphology and specific elemental composition are consistent with the higher corrosion resistance observed for the 1500-cycle samples.
10. The FTIR analyses confirmed that the primary corrosion reactions were associated with the formation of Mg(OH)_2_ and subsequently MgCO_3_, and then ZnO and MgO.
11. The presence of Zn was revealed in the phase analysis of corrosion products on the MgCa2Zn1Gd3 alloy with the ZnO layer deposited after 1500 cycles, which may suggest the presence of a ZnO coating after 100 h of immersion in a high-chloride solution.
12. Consequently, it can be concluded that the zinc oxide film deposited after 1500 cycles in the MgCa2Zn1Gd3 alloy is a highly promising and viable option for improving the corrosion resistance of biodegradable magnesium implants, as demonstrated by the findings described above.

## Figures and Tables

**Table 1:** Surface roughness parameters of the thin ZnO on MgCa4Zn1Gd1 and MgCa2Zn1Gd3 alloys.

Substrate	ZnO (Deposition Cycles)	Roughness Average, R_a_, nm	Root Mean Square, R_S_, nm
MgCa4Zn1Gd1	MgCa4Zn1Gd1	7.06 ± 0.05	8.76 ± 0.05
	ZnO (500 cycles)	12.12 ± 0.05	15.69 ± 0.05
	ZnO (1000 cycles)	10.83 ± 0.05	13.76 ± 0.05
	ZnO (1500 cycles)	10.40 ± 0.05	13.49 ± 0.05
MgCa2Zn1Gd3	MgCa2Zn1Gd3	5.76 ± 0.05	7.48 ± 0.05
	ZnO (500 cycles)	15.09 ± 0.05	18.52 ± 0.05
	ZnO (1000 cycles)	14.69 ± 0.05	17.90 ± 0.05
	ZnO (1500 cycles)	7.65 ± 0.05	9.80 ± 0.05

**Table 2:** Electrochemical corrosion parameters of ZnO thin films and the substrate Mg alloys.

Sample	Corrosion Potential, E_corr_, V	Polarization Resistance, R_p_, Ω·cm^2^	Corrosion Current Density, j_corr_, mA·cm^−2^
MgCa4Zn1Gd1	−1.652	900	58.6 × 10^−3^
MgCa4Zn1Gd1/ZnO (500)	−1.618	980	51.1 × 10^−3^
MgCa4Zn1Gd1/ZnO (1000)	−1.596	1200	45.3 × 10^−3^
MgCa4Zn1Gd1/ZnO (1500)	−1.587	1800	29.2 × 10^−3^
MgCa2Zn1Gd3	−1.651	903	41 × 10^−3^
MgCa2Zn1Gd3/ZnO (500)	−1.598	1311	23.2 × 10^−3^
MgCa2Zn1Gd3/ZnO (1000)	−1.577	47,060	1.1 × 10^−3^
MgCa2Zn1Gd3/ZnO (1500)	−1.562	59,480	0.7 × 10^−3^

**Table 3:** Corrosion rates of ZnO thin films, and the MgCa4Zn1Gd1 and MgCa2Zn1Gd3 alloys after 100 h of immersion in Ringer solution at 37 °C.

Sample	Corrosion Rate, CR, mg/cm^2^·Day
MgCa4Zn1Gd1	2.12
MgCa4Zn1Gd1/ZnO (500 cycles)	1.16
MgCa4Zn1Gd1/ZnO (1000 cycles)	0.58
MgCa4Zn1Gd1/ZnO (1500 cycles)	0.23
MgCa2Zn1Gd3	1.26
MgCa2Zn1Gd3/ZnO (500 cycles)	0.63
MgCa2Zn1Gd3/ZnO (1000 cycles)	0.42
MgCa2Zn1Gd3/ZnO (1500 cycles)	0.21

## Data Availability

The original contributions presented in this study are included in the article. Further inquiries can be directed to the corresponding author.
